# Exopolysaccharide-Producing Bacteria Regulate Soil Aggregates and Bacterial Communities to Inhibit the Uptake of Cadmium and Lead by Lettuce

**DOI:** 10.3390/microorganisms12112112

**Published:** 2024-10-22

**Authors:** Heyun Zhang, Ke Wang, Xinru Liu, Lunguang Yao, Zhaojin Chen, Hui Han

**Affiliations:** 1 Collaborative Innovation Center of Water Security for Water Source Region of Mid-route Project of South-North Water Diversion of Henan Province, Nanyang Normal University, Nanyang 473061, China; zhangheyun66@163.com (H.Z.); m13080182307@163.com (K.W.); 17651962022@163.com (X.L.); 2 Henan Field Observation and Research Station of Headwork Wetland Ecosystem of the Central Route of South-to-North Water Diversion Project, Nanyang Normal University, Nanyang 473061, China; lunguangyao@nynu.edu.cn

**Keywords:** immobilization, exopolysaccharides, microaggregates, heavy metal, antioxidant enzyme, nitrogen cycling

## Abstract

The accumulation of heavy metals in the soil not only causes serious damage to the soil ecosystem, but also threatens human health through the food chain. Exopolysaccharides have the functions of adsorbing and chelating heavy metals and reducing their bioavailability in the soil. In our study, exopolysaccharide-producing bacteria with a high efficiency in adsorbing cadmium (Cd) and lead (Pb) were screened from heavy metal-contaminated farmland. Through pot experiments, the influence of functional strains on the size distribution, heavy metal content, and bacterial community structure of soil aggregates in lettuce was studied using high-throughput sequencing technology. The results show that 11 strains secreting exopolysaccharides were initially screened from heavy metal-contaminated soil. Among them, strain Z23 had a removal rate of 88.6% for Cd and 93.2% for Pb. The rate at which Cd was removed by strain Z39 was 92.3%, and the rate at which Pb was removed was 94.4%. Both strains belong to *Bacillus* sp. Strains Z23 and Z39 induced the formation of Fe_2_Pb(PO_4_)_2_, Cd_2_(PO_4_)_2_, and Pb_2_O_3_ in the solution. The pot experiments showed that strains Z23 and Z39 increased (19.1~23.9%) the dry weight and antioxidant enzyme activity of lettuce roots and leaves, while reducing (40.1~61.7%) the content of Cd and Pb. Strains Z23 and Z39 increased the proportion of microaggregates (<0.25 mm) and the content of exopolysaccharides in rhizosphere soil and reduced (38.4–59.7%) the contents of available Cd and Pb in microaggregates, thus inhibiting the absorption of heavy metals by lettuce. In addition, the exopolysaccharide content and the bacterial community associated with heavy metal resistance and nitrogen (N) cycling (*Patescibacteria*, *Saccharimonadales*, *Microvirga*, and *Pseudomonas*) in microaggregates were key factors affecting the available heavy metal content in soil. These results show that the exopolysaccharide-producing bacteria Z23 and Z39 reduced the absorption of Cd and Pb by lettuce tissues, thus providing strain resources for the safe utilization of soils that exceed heavy metal standards for farmland and for reducing the heavy metal content in vegetables.

## 1. Introduction

Due to industrial production activities, such as mining and gold plating or the improper disposal of agricultural waste, heavy metal pollution has occurred in some farmland [1]. Cadmium (Cd) and lead (Pb) are among the most toxic environmental pollutants, are easily enriched in agricultural soils, have strong migration capabilities, and cannot be degraded by microorganisms [2,3]. They are absorbed by crops and ultimately enter the human body through the food chain and pose potential risks to human health [4]. For example, chronic Cd poisoning causes kidney damage, mainly involving renal tubule lesions. In severe cases, chronic renal failure, osteomalacia, and osteoporosis may occur [5]. Currently, there are several methods to prevent crops from absorbing heavy metals from the soil, such as screening crop varieties, in situ passivation remediation, agronomic measures (deep dive and water and fertilizer management), and foliar fertilizer spraying [6]. Among them, in situ passivation remediation is a feasible and effective method. The most important aspect of in situ passivation remediation technology is the screening of passivators, which involves compost, straw, lime, clay minerals, and functional microorganisms [7,8]. Compared to chemical passivators, such as lime and clay minerals, microorganism passivators have inevitably lived in heavy metal-contaminated soil for a long time, thus demonstrating strong resistance to heavy metals and exhibiting corresponding detoxification mechanisms [9]. At the same time, they also play a certain role in controlling the interaction with crop roots [10]. These microorganisms immobilize heavy metals in the soil, reduce their toxicity, and secrete substances such as indole acetic acid (IAA), siderophores, polyamines, and arginine to promote plant growth, improve crop quality, and improve soil quality [11,12].

Exopolysaccharides are metabolic products secreted by microorganisms during growth and metabolism and are easily separated from the bacterial cell wall [13]. They are a type of macromolecular substance that has good adsorption and passivation effects on heavy metals. Exopolysaccharides adsorb organic nutrients in the external environment, provide energy for cell metabolism, and adsorb inorganic ions, including heavy metal ions, effectively promoting the formation of exopolysaccharide gel-like substances and minerals [14]. The mechanisms by which microorganisms use exopolysaccharides to adsorb heavy metals mainly involve surface complexation and ion exchange [15]. The exopolysaccharides produced by biofilms can also be used to remove aluminum (Al), Pb, zinc (Zn), and nickel (Ni) [16]. Copper (Cu) binds to hydroxyl and phosphoryl groups and Al binds to amino and carbonyl groups in exopolysaccharides [17]. Pb^2+^ can be removed because it chelates with carboxyl, hydroxyl, and C-O-C groups in exopolysaccharides [18].

In nature, there are many kinds of microorganisms that can synthesize exopolysaccharides, such as bacteria, fungi, and actinomycetes [19]. According to statistics, 76 species of microorganisms belonging to 49 genera have been found to produce exopolysaccharides, among which the main exopolysaccharide-producing bacteria are *Bacillus*, *Pseudomonas*, *Streptococcus*, and *Lactobacillus* [20,21]. Due to the important role of exopolysaccharides in the field of heavy metal pollution bioremediation, it has become a research focus at home and abroad. *Halomonas* sp. produces exopolysaccharides under arsenic (As) stress, which oxidizes toxic As^3+^ to As^6+^ of lower toxicity, thereby detoxifying or bioremediating As in the soil and promoting the growth of rice under As stress [22]. The mechanisms by which exopolysaccharide-producing bacteria adsorb heavy metals mainly include complexation, ion exchange, biological precipitation, and oxidation–reduction [23,24]. Although some scholars have reported the use of exopolysaccharide-producing bacteria to immobilize heavy metals, the mechanisms used by these bacteria in immobilizing Cd and Pb are not well understood. In addition, there are few studies on the effects of exopolysaccharide-producing bacteria isolated from heavy metal-contaminated soil on the absorption of Cd and Pb by lettuce [25].

It is assumed that exopolysaccharide-producing bacteria can immobilize Cd and Pb and inhibit the absorption of heavy metals by vegetables. However, the soil and microbiological mechanisms used by exopolysaccharide-producing bacteria in inhibiting the absorption of Cd and Pb by lettuce are still unclear. Therefore, rhizosphere soils were collected from Cd- and Pb-contaminated fields and exopolysaccharide-producing strains were screened according to the exopolysaccharide production medium. The mechanisms of Cd and Pb adsorption used by functional strains were studied by means of scanning electron microscopy (SEM) and X-ray photoelectron spectroscopy (XPS). Moreover, the effect of functional strains on the growth and heavy metal absorption in lettuce and its soil and microbial mechanisms were studied through pot experiments. The research results are expected to provide strain resources and technical support for implementing heavy metal-contaminated farmland bioremediation and ensuring crop safety.

## 2. Materials and Methods

### 2.1. Screening of Exopolysaccharide-Producing Bacteria

Cd-contaminated soil was collected from vegetable roots in the suburbs of Jiyuan city, Henan Province, China (35°03′ N, 112°61′ E), and brought back to the laboratory. Then, the rhizosphere soil (2 g) was weighed and placed into a shaker containing 50 mL sterile deionized water, and shaken at 30 °C for 2 h. The culture solution (1 mL) was placed into a test tube containing 9 mL of sterile deionized water, and a pipette was used to blow the evenly mixed solution into 10^−1^ diluent. A pipette was used to draw 1 mL of 10^−1^ diluent, which was then poured into another test tube containing 9 mL of sterile deionized water to form 10^−2^ diluent, and finally to form 10^−3^, 10^−4^, and 10^−5^ soil diluents. Then, 0.5 mL diluents of different gradients were absorbed and evenly coated on solid nitrogen medium (sucrose 10.0 g, K_2_HPO_4_ 2.0 g, MgSO_4_ 7H_2_O 0.5 g, yeast powder 0.5 g, NaCl 0.1 g, agar powder 20.0 g, deionized water 1000 mL, pH 7.0, Shanghai McLean Biochemical Technology Co., Ltd., Shanghai, China) on a plate and incubated at 30 °C for 3 days in a biochemical incubator (Shengwei-SPXD300, Shanghai, China). A sterilized toothpick was used to pick out the colonies with a large amount of mucus on the surface, and a single colony was obtained through four zoning lines and labeled.

### 2.2. Determination of the Ability of Exopolysaccharide-Producing Bacteria to Adsorb Heavy Metals

A shake flask containing 50 mL of nitrogen-containing liquid medium was filled with Cd and Pb mother liquor, resulting in a Cd concentration of 5 mg L^−1^ and a Pb concentration of 10 mg L^−1^ in the flask. The bacterial suspension of the selected strains was inoculated at a ratio of 1% and cultured for 3 days at 28 °C with shaking. Each strain experiment was repeated three times [26]. After the culture was completed, 5 mL of culture solution was taken, centrifuged at 7000 r min^−1^ for 10 min in a constant temperature shaker (PY180S, Huicheng, China), and the supernatant was collected for digestion on an electric hot plate. The concentrations of Cd and Pb in the supernatant were measured using an inductively coupled plasma optical emission spectrometer (ICP-OES, Shimadau-ICPE 9820, Shanghai, China), and the removal rate of Cd and Pb by the strain was calculated. The content of polysaccharides in the culture solution was determined using sulfuric acid–anthrone colorimetry. Briefly, 1 mL of the aforementioned supernatant was added to 3 mL of absolute ethanol, and the liquid in the centrifuge tube was tapped with a pipette until white flocculent precipitate appeared. The centrifuge tube was covered and placed in a 4 °C refrigerator for 24 h, and then centrifuged at 7000 r min^−1^ for 10 min. The supernatant was decanted, dried in a 37 °C oven, and added to 1 mL of deionized water and 0.5 mL of Sevage reagent (chloroform/n-butanol = 4:1). The mixture was inverted and mixed evenly, and then left to stand for 1 h at 37 °C, followed by centrifugation at 7000 r min^−1^ for 10 min. The supernatant was decanted and added to 5 mL of sulfuric acid anthrone, heated in a 100 °C water bath for 10 min, and then the absorbance at 620 nm was measured using a spectrophotometer (UV762PC, Nanjing, Xuxi, China). The polysaccharide content was calculated using a standard curve [27]. The strains with the highest ability to remove Cd and Pb and secrete polysaccharides were the target strains for further study.

### 2.3. Determination of Biological Characteristics of Functional Strains

The DNA of the strains was extracted and genus identification was performed using 16S rDNA sequencing [28]. Solid nitrogen-containing culture plates containing different concentrations of Cd (100–800 mg L^−1^) and Pb (120–2000 mg L^−1^) were prepared to determine the resistance of the target strains to Cd and Pb. The indoleacetic acid (IAA) produced by the strains was quantified using the methods described by Jiang et al. [29]. The siderophores produced by the strains were quantified using the chrome azurol-s (CAS) analytical method [30]. The ability of the target strains to produce 1-aminocyclopropane-1-carboxylate (ACC) deaminase was determined according to the method used by Belimov et al. [31]. Gram staining was used to identify the positive and negative strains.

### 2.4. Adsorption of Cd and Pb by Exopolysaccharide-Producing Bacteria

The mother solution of Cd and Pb was added to a shake flask containing 50 mL of nitrogen-containing liquid medium, resulting in a Cd concentration of 5 mg L^−1^ and a Pb concentration of 10 mg L^−1^ in the flask. The target bacterial suspension was inoculated at a ratio of 1% into the flask, and the flask was incubated at 28 °C for 7 days. There were three treatments: the solution containing Cd and Pb (CK), the solution containing both bacterial strains and heavy metals, and the solution containing only bacterial strains. Each treatment had three replicates. On days 0, 1, 3, 5, and 7, a portion of the culture broth was taken, centrifuged at 7000 r min^−1^ for 10 min, and the supernatant was collected. The concentrations of Cd and Pb in the solution were determined using ICP-OES, the pH of the supernatant was measured using a pH meter (PHS-3CT, Jingsheng, Shanghai, China), and the polysaccharide content in the supernatant was determined using sulfuric acid–anthrone colorimetry.

### 2.5. Mechanism of Exopolysaccharide-Producing Bacteria Adsorbing Cd and Pb

The cell pellets of the 7th day of the adsorption experiment were collected and then mixed with 15 mL of 2.5% (*v*/*v*) glutaraldehyde. They were left to stand at 4 °C for 4 h and then centrifuged at 5000 r min^−1^ for 10 min. The supernatant was removed. Following this, 50%, 75%, 90%, and 100% ethanol solutions were added in order, and the solution was left to stand for 10 min at room temperature. Then, the solution was centrifuged at 5000 r min^−1^ for 10 min, and the pellets were placed in a freeze dryer for 48 h [32]. The morphology of the samples was characterized using a field emission scanning electron microscope–energy spectrometer (SEM-EDS, Hitachi I-SU8010, Dongjing, Japan). The surface functional groups of the samples were characterized using a Fourier-transform infrared spectrometer (FIRT, America Thermo Fisher Scientific-iS50, Shanghai, China). The composition of the sediment on the surface of the cells was determined using an X-ray diffractometer (XRD, D-MAX2500, Japan Corporation Co., Ltd., Tokyo, Japan). The typical elemental valence state changes on the surface of the cells were analyzed using an X-ray photoelectron spectrometer (XPS, America Thermo Fisher Scientific K-Alpha, Shanghai, China).

### 2.6. Pot Experiment with Lettuce

Soil samples were collected (35°03′ N, 112°61′ E), naturally air-dried, and placed in pots, with each pot containing 3 kg of soil (Cd 2.45 mg kg^−1^; Pb 179 mg kg^−1^; pH 6.92; organic matter 27.9 g kg^−1^, total N 1.74 g kg^−1^, available P 0.48 g kg^−1^, total P 1.41 g kg^−1^, total K 1.32 g kg^−1^, and available K 1.11 g kg^−1^). Lettuce seeds were soaked in 75% ethanol for 3 min, washed with deionized water 3 times, evenly sprinkled in the pots, and covered with fine soil. After the lettuce sprouted, the seedlings were gradually thinned out, with 5 seedlings per pot. A total of 3 treatments were set up in the experiment: the control group (CK), the Z23 treatment group (Z23), and the Z39 treatment group (Z39), with 4 replicates for each treatment. Among all of the screened strains, Z23 and Z39 had the strongest ability to remove Cd and Pb, which were used for this part of the experiment. When the lettuce grew into 4 small leaves, the strains were inoculated. The strains Z23 and Z39 were activated and prepared into a bacterial suspension (10^8^ CFU mL^−1^). A small ditch was drawn around the lettuce, the bacterial solution was poured in at an amount of 25 mL (10^8^ CFU mL^−1^) per pot, and the control was replaced with sterile deionized water. After inoculating the strains, the cultivation was continued for 30 days, and then the harvesting process was carried out.

### 2.7. Effects of Exopolysaccharide-Producing Bacteria on Lettuce Growth and Cd Uptake

The roots and leaves of lettuce were separated using scissors, and the roots were soaked in a 0.01 mol L^−1^ EDTA-2Na solution for 10 min and rinsed with deionized water. Fresh lettuce roots and leaves were taken, and the activities of superoxide dismutase (SOD) in the samples were determined using a SOD assay kit (A001–1–1, Nanjing Jiancheng Bioengineering Institute, Nanjing, China). The activities of peroxidase (POD) in the samples were determined using a POD assay kit (A084–3–1, Nanjing Jiancheng Bioengineering Institute, Nanjing, China). Three randomly selected lettuce leaves and roots were placed in a file bag and labeled, and then dried in an oven at 80 °C until constant weight and measured for dry weight. The dried lettuce leaves and roots were cut with scissors; weighed to 0.1 g; added to a crucible with 3 mL of HNO_3_ (65%), 1 mL of HCl (36%), 2 mL of HClO_4_ (70%), and 2 mL of HF (42%); and left for 18 h before digestion. Finally, the contents of Cd and Pb in the digestion solution were determined using ICP-OES [33], and the contents of Cd and Pb in the lettuce tissue were calculated.

### 2.8. Effects of Exopolysaccharide-Producing Bacteria on the Structure of Rhizosphere Soil Aggregates

Rhizosphere soil was collected from the rhizosphere of the lettuce, allowed to dry naturally without grinding, and then placed directly on a sieve (with a pore size of 0.25 mm) to collect soil aggregates of two particle sizes: large aggregates (>0.25 mm) and microaggregates (<0.25 mm) [34]. The content of DTPA-Cd and DTPA-Pb in the large aggregates and microaggregates was determined using ICP-OES [33]. The content of polysaccharides was determined using sulfuric acid–anthrone colorimetry. The total potassium (TK), total phosphorus (TP), ammoniacal nitrogen (NO_4_^+^), and nitric nitrogen (NO_3_^−^) contents in the rhizosphere soils of the lettuce were detected via the methods described by Han et al. [35].

### 2.9. Determination of Bacterial Community Structure in Soil Aggregates

The total DNA of rhizosphere soil large aggregates and microaggregates in the CK, Z23, and Z39 treatment groups was extracted. Then, PCR amplification of the V3–V4 variable region of the total DNA was performed [36]. A PE 2*300 library was constructed using the purified amplification fragments according to the standard operating procedures of the Illumina MiSeq platform (Illumina, San Diego, CA, USA). For each cluster file, the diversity (Sobs, Chao1, Simpson, and Shannon and PD indices) was calculated with MOTHUR. The analysis project was assisted by the Shanghai Meiji Company, Shanghai, China (https://www.majorbio.com/, accessed on 8 August 2023).

### 2.10. Data Analysis

Excel 2010, SPSS 17.0, Avantage 6.6, and Omnic 8.2 software were used for data analysis and statistics. The mathematically processed results are presented in the form of M ± SE, where M is the arithmetic mean and SE is the standard error (n = 4). All the data were subjected to homogeneity testing before Tukey’s multiple comparison. One-way ANOVA and Tukey’s test (*p* < 0.05) were used to compare the heavy metal removal rate, pH, and polysaccharide contents of the solution; the Cd and Pb contents in the lettuce plants; and the SOD and POD activities in the fresh lettuce. Two-factor ANOVA was used to compare dry weights of lettuce plants, the DTPA-extractable Cd and Pb contents in the rhizosphere soils of the lettuce, and the polysaccharide contents in the soils of the lettuce. Redundancy analysis (RDA) was performed using CANOCO 4.5 to examine the relationship between the soil quality and the DTPA-extractable Cd and Pb contents.

## 3. Results

### 3.1. Screening of Exopolysaccharide-Producing Bacteria with Heavy Metal Adsorption Capacity

**Figure 1:**
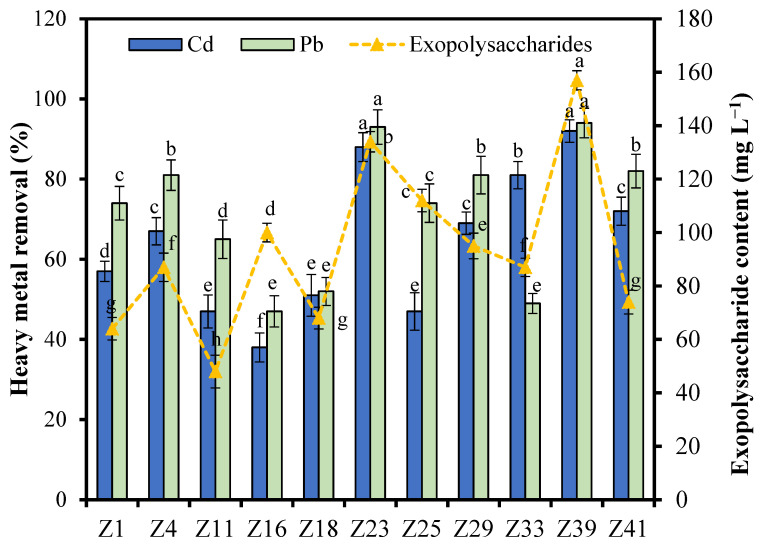
The ability of strains to remove Cd and Pb and produce exopolysaccharides. Error bars are ±standard error (n = 4). Different lowercase letters (a–h) for the same test indicator indicate significant (*p* < 0.05) differences between treatments according to Tukey’s test.

The exopolysaccharide-producing bacteria were initially screened from heavy metal-contaminated rhizosphere soil using an exopolysaccharide-producing medium, and 11 stable exopolysaccharide-producing bacterial strains were finally selected. The removal rates of Cd for these 11 strains ranged from 38.6% to 92.3%, and the removal rates for Pb ranged from 49.2% to 94.4%. Among them, strain Z23 had a removal rate of 88.6% for Cd and 93.2% for Pb. Strain Z39 had a removal rate of 92.3% for Cd and 94.4% for Pb (Figure 1). Subsequently, the exopolysaccharide-producing abilities of these 11 bacterial strains were determined, and it was found that the polysaccharide content ranged from 64.7 mg L^−1^ to 157 mg L^−1^, with strain Z23 producing 134 mg L^−1^ of polysaccharide and strain Z39 producing 157 mg L^−1^ of polysaccharide (Figure 1). Comprehensive analysis showed that strains Z23 and Z39 had high adsorption and removal abilities for Cd and Pb as well as polysaccharide production and could be used as test strains for further research.

### 3.2. Biological Characteristics of the Target Bacterial Strain

The total DNA of the tested strains Z23 and Z39 was used as a template for 16S rDNA amplification and sequencing. The 16S rDNA sequence of strain Z23 was 100% similar to that of *Bacillus paramobilis* BML-BC017, and it was preliminarily identified as *Bacillus* sp. (Figure S1). The 16S rDNA sequence of strain Z39 was 100% similar to that of *Bacillus altitudinis* 41KF2b, and it was preliminarily identified as *Bacillus* sp. (Figure S1). In general, strains Z23 and Z39 belong to the genus *Bacillus*. Strains Z23 and Z39 had the ability to secrete high concentrations of IAA, siderophores, and ACC polyaminase, indicating that both strains had the ability to promote crop growth (Table S1). The lethal concentration of Cd for strain Z23 was 400 mg L^−1^, and the lethal concentration of Pb was 1800 mg L^−1^. The lethal concentration of Cd for strain Z39 was 500 mg L^−1^, and the lethal concentration of Pb was 2000 mg L^−1^ (Table S1). These results indicated that both strains had high resistance to heavy metals and survived well in contaminated environments, providing possibilities for the subsequent remediation of heavy metal-contaminated soil.

### 3.3. Adsorption of Cd and Pb by Functional Strains

**Figure 2:**
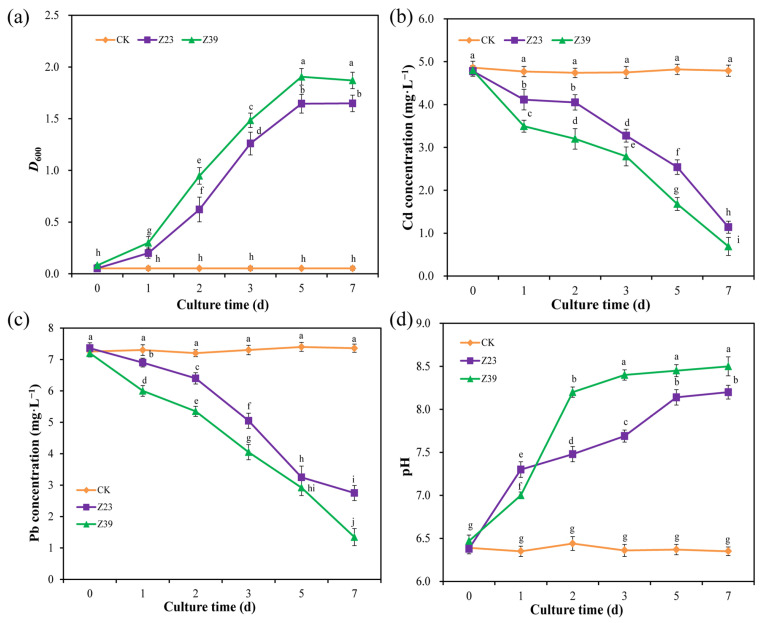
Effects of functional strains on the concentration of heavy metals in the culture solution. (**a**) OD_600_; (**b**) Cd concentration (mg L^−1^); (**c**) Pb concentration (mg L^−1^); (**d**) pH. Error bars are ±standard error (n = 4). Bars indicated by the same letter were not significantly (*p* > 0.05) different according to Tukey’s test.

The optical density in 600 nm (OD_600_) of strains Z23 and Z39 in solutions with Cd (5 mg L^−1^) and Pb (10 mg L^−1^) gradually increased with the increase in the culture time (Figure 2a). When strains Z23 and Z39 were cultured for 7 days, the OD_600_ in the solution reached 1.65 and 1.87, respectively, indicating that the viable strains Z23 and Z39 were able to grow normally under the stress of Cd and Pb. The concentration of Cd in the CK solution remained between 4.79 mg L^−1^ and 4.86 mg L^−1^, while that in the Z23 and Z39 solutions gradually decreased with an increase in the culture time. The Cd removal rate reached 76.2% and 85.6% on day 7 of culture, respectively (Figure 2b), indicating that strains Z23 and Z39 had strong adsorption capacity for Cd. As the culture days increased, the concentration of Pb in the CK solution remained between 7.25 mg L^−1^ and 7.35 mg L^−1^. The concentration of Pb in the Z23 and Z39 solutions gradually decreased, with the Pb reaching 2.75 mg L^−1^ in the Z23 solution and 1.35 mg L^−1^ in the Z39 solution on day 7 of culture (Figure 2c). The pH in the CK solution remained unchanged (6.48), while both Z23 and Z39 significantly increased the pH of the solution (Figure 2d). On day 7 of culture, the pH in the Z23 solution was 8.23 and in the Z39 solution was 8.51, indicating that strains Z23 and Z39 increased the pH of the solution and thereby reduced the concentration of Cd and Pb.

### 3.4. Mechanism of Functional Strains Adsorbing Cd and Pb

**Figure 3:**
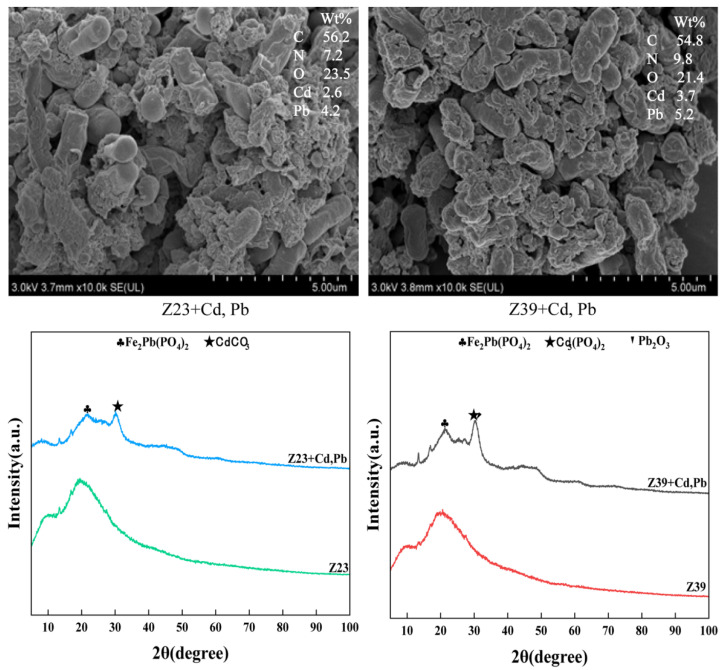
SEM-EDS and XRD images of functional strains adsorbing heavy metals.

The cell pellets of strains Z23 and Z39 were collected, and the SEM images showed that there were many precipitate particles on the cell walls of strains Z23 and Z39, while EDS analysis showed that these precipitate particles contained Cd and Pb (Figure 3), indicating that the cell walls of strains Z23 and Z39 immobilized Cd and Pb. A comparison with standard cards revealed that some compounds containing Cd and Pb were observed on the cell walls of the tested strains during the process of Z23 and Z39’s immobilization of heavy metals, including iron(II) lead(II) phosphate (Fe_2_Pb(PO_4_)_2_), cadmium phosphate (Cd_3_(PO_4_)_2_), and lead sesquioxide (Pb_2_O_3_) (Figure 3). Moreover, the synthesized compounds varied among the different strains. In strain Z23, Fe_2_Pb(PO_4_)_2_ and Cd_3_(PO_4_)_2_ were detected, while in strain Z39, Fe_2_Pb(PO_4_)_2_, Cd_3_(PO_4_)_2_, and Pb_2_O_3_ were detected. Therefore, strains Z23 and Z39 induced the formation of precipitates of Cd and Pb, thereby reducing the content of Cd and Pb in the solution. When synthesizing Cd and Pb precipitates, the types of precipitates synthesized on the cell surface of strain Z39 were more diverse than those of strain Z23, which was consistent with the adsorption rate results of Cd and Pb for the strains. The adsorption and removal effect of Cd and Pb by strain Z39 was slightly better than that of strain Z23.

### 3.5. XPS Analysis

**Figure 4:**
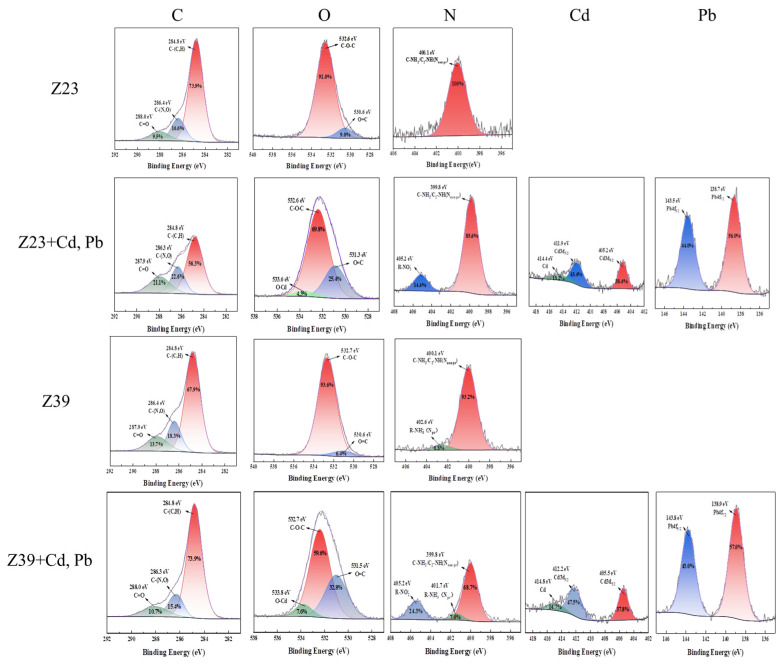
XPS images of functional strains adsorbing heavy metals.

The XPS spectrum analysis of the samples showed changes in the main elements carbon (C), oxygen (O), nitrogen (N), Cd, and Pb (Figure 4). It was found that the peak intensities of C and O elements were higher, indicating that EPS was mainly composed of C and O elements. C showed three types of peaks: carbon–carbon single bond/carbon–hydrogen bond (C-C/C-H) at 284.8 eV, carbon–oxygen single bond/carbon–nitrogen single bond (C-O/C-N) at 286.3–286.54 eV, and carbon–oxygen double bond (C=O) at 287.9–288 eV. Under the stress of Cd and Pb, the content of C-(C, H) on the surface of the bacteria decreased, while the content of C-(N, O) and C=O increased, indicating that C-(C, H) fracture occurred in the C element in EPS. C combined with O or N to form C-N or C=O and existed in proteins, indicating that the strain can produce protein-rich EPS after heavy metal stress, and the increase in O or N functional groups with a C structure improved the heavy metal removal ability of EPS [37]. The content of C-O-C decreased, while the content of C=O increased, and a new O-Cd peak appeared (533.6–533.8 eV), indicating that part of the C-O was converted to C=O. The increase in C=O was conducive to the adsorption of metals by EPS [38]. There were two types of N in the EPS of the strains without Cd and Pb: C-NH_2_/C_2_-NH at 399.8–400.1 eV, and R-NH^3+^ at 401.7–402.86 eV. Under Cd and Pb stress, the contents of C-NH_2_/C_2_-NH and R-NH^3+^ in the EPS decreased and R-NO^2−^ was produced, indicating that C-NH_2_/C_2_-NH was converted into R-NO^2^ groups, which could bind to heavy metals [39]. In addition, new strong characteristic peaks of Cd, Cd3d_3/2_, Cd3d_5/2_, Pb4f_5/2_, and Pb4f_7/2_ appeared on the cell surface, indicating that heavy metals had been successfully adsorbed on the cell surfaces of strains Z39 and Z23.

### 3.6. Dry Weight, Heavy Metal Content, and Antioxidant Enzyme Activity in Lettuce

**Figure 5:**
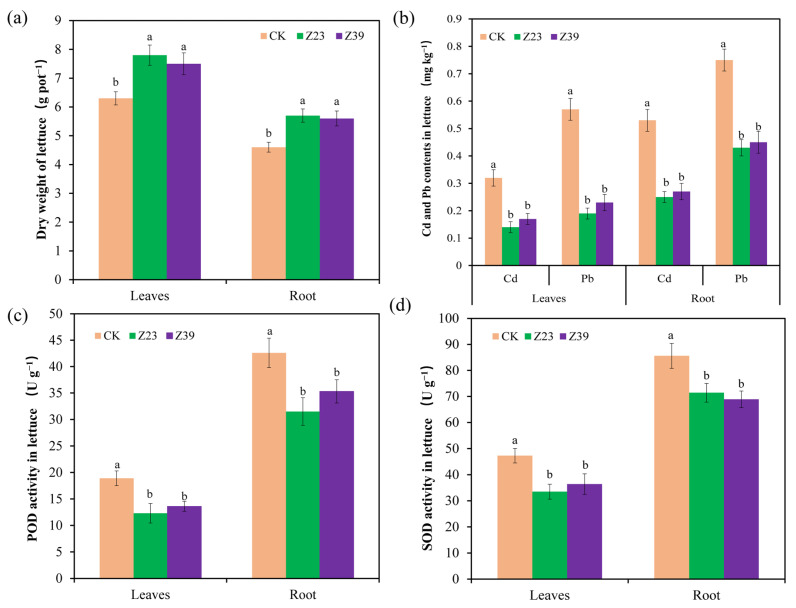
Effects of different treatments on the dry weight, Cd and Pb contents, and antioxidant enzymes of lettuce. (**a**) Dry weight of lettuce; (**b**) Cd and Pb contents in lettuce; (**c**) POD activity; (**d**) SOD activity. Error bars are ±standard error (n = 4). Bars indicated by the same letter were not significantly (*p* > 0.05) different according to Tukey’s test.

Compared with the control, inoculation with strains Z23 and Z39 significantly increased the dry weight of lettuce roots and leaves by 19.1% to 23.9% (Figure 5a). The contents of Cd and Pb in the lettuce roots in the CK treatment were 0.53 mg kg^−1^ and 0.75 mg kg^−1^, respectively (Figure 5b). However, the inoculation of strain Z23 decreased the contents of Cd and Pb in the lettuce roots by 52.8% and 42.7%, respectively. The inoculation of strain Z39 decreased the contents of Cd and Pb in the lettuce roots by 49.1% and 40.1%, respectively (Figure 5b). Moreover, the contents of Cd and Pb in the lettuce leaves were 0.32 mg kg^−1^ and 0.57 mg kg^−1^ in the CK treatment. Strain Z23 decreased the contents of Cd and Pb in the lettuce leaves by 52.8% and 42.7%, respectively. Strain Z39 decreased the contents of Cd and Pb in the lettuce leaves by 52.8% and 42.7%, respectively (Figure 5b). The POD activity values in the lettuce roots and leaves were 42.6 U g^−1^ and 18.9 U g^−1^, respectively, in the CK treatment; however, the inoculation of strains Z23 and Z39 decreased the POD activity in the lettuce roots and leaves by 17.1% to 34.9% (Figure 5c), indicating that these strains reduced the toxicity of Cd and Pb in lettuce, leading to a decrease in POD activity in lettuce tissues. Similarly, the SOD activity values in lettuce roots and leaves in the CK treatment were 85.6 U g^−1^ and 47.3 U g^−1^, respectively. The inoculation of strains Z23 and Z39 also significantly decreased the SOD activity in lettuce roots by 16.5% to 29.1% (Figure 5d), indicating that the inoculation of strains Z23 and Z39 reduces the toxicity of Cd and Pb in lettuce, leading to a decrease in SOD activity in lettuce tissues.

### 3.7. Particle Size Distribution and Polysaccharide Content in Soil Aggregates

**Figure 6:**
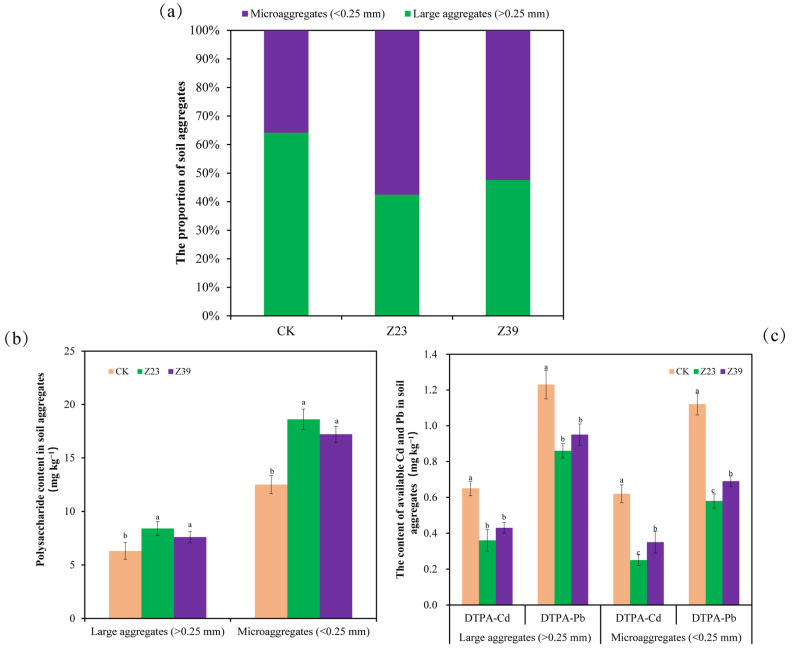
Effects of exopolysaccharide-producing bacteria on the particle size distribution and exopolysaccharide content of soil aggregates in the rhizosphere soil of lettuce. (**a**) Proportion of different particle size aggregates. (**b**) Exopolysaccharide content. (**c**) Contents of available heavy metals in lettuce rhizosphere soil. Error bars are ±standard error (n = 4). Bars indicated by the same letter were not significantly (*p* > 0.05) different according to Tukey’s test.

The polysaccharide content in soil can affect the distribution of aggregate size and the mobility of heavy metals [40]. In the CK control group, the proportion of large aggregates (>0.25 mm) in the rhizosphere soil was 64.2%, and the proportion of microaggregates (<0.25 mm) was 35.8% (Figure 6a). The proportion of large aggregates in the rhizosphere soil was 42.5%, and the proportion of microaggregates was 57.5% in the Z23 treatment. In the treatment group inoculated with Z39, the proportion of large aggregates in the rhizosphere soil was 47.6%, and the proportion of microaggregates was 52.4% (Figure 6a). This indicated that strains Z23 and Z39 increased the proportion of microaggregates in the lettuce rhizosphere soil, thereby enhancing the ability of the soil to adsorb and immobilize Cd and Pb [39]. Further research showed that both Z23 and Z39 significantly increased (20.6–48.8%) the polysaccharide content in the rhizosphere soil compared to the CK control, especially in microaggregates (Figure 6b), indicating that microaggregates play an important role in immobilizing heavy metals. The content of DTPA-Cd in large aggregates was 0.65 mg kg^−1^ and the content of DTPA-Pb was 1.23 mg kg^−1^ in the CK group (Figure 6c). However, the contents of DTPA-Cd and DTPA-Pb in large aggregates in the treatment groups of strains Z23 and Z39 significantly decreased (22.8–44.6%) (Figure 6c). Similarly, in the lettuce rhizosphere soil of the CK group, the content of DTPA-Cd in microaggregates was 0.62 mg kg^−1^ and the content of DTPA-Pb was 1.12 mg kg^−1^. However, the content of DTPA-Cd and DTPA-Pb in microaggregates in the treatment groups of strains Z23 and Z39 also significantly decreased (38.4–59.7%) (Figure 6c). These results showed that inoculating the polysaccharide-producing bacteria Z23 and Z39 reduced the content of available heavy metals in the rhizosphere soil, thereby preventing the lettuce from absorbing heavy metals.

### 3.8. Bacterial Community Structure and Diversity of Soil Aggregates

**Figure 7:**
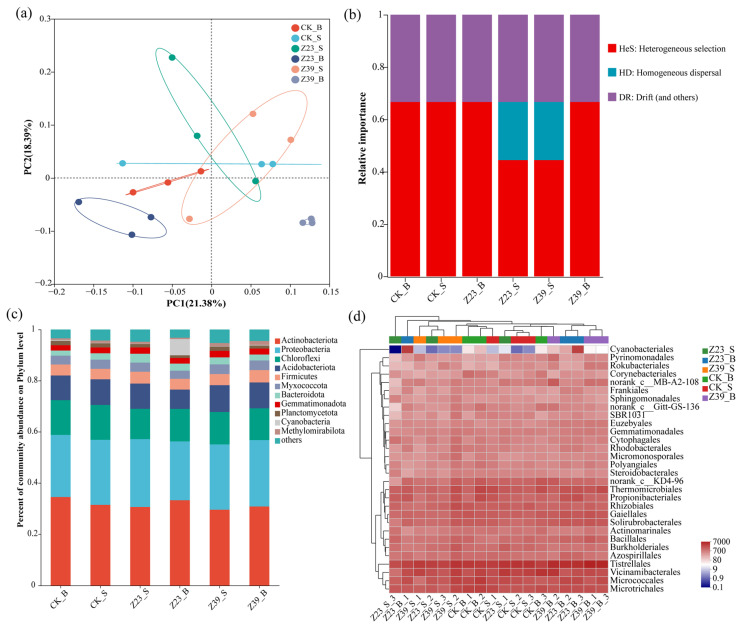
Effects of strains Z23 and Z39 on the bacterial community diversity of rhizospheric soil aggregates. (**a**) PCoA. (**b**) Analysis of βNTI community structure. (**c**) Relative abundances of sequences at the phylum level. (**d**) Relative abundances of sequences at the order level.

The changes in bacterial community structure were analyzed for large aggregate soil and microaggregate soil in the CK treatment group (CK-B and CK-S), the Z23 treatment group (Z23-B and Z23-S), and the Z39 treatment group (Z39-B and Z39-S). The PCoA results showed that the samples treated differently were dispersed, large aggregate samples were clustered together, and microaggregate samples were clustered together (Figure 7a), indicating that the bacterial community structure of the large aggregate samples was different from that of the microaggregate samples. The Alpha diversity results also indicated that inoculating strains Z23 and Z39 could alter the bacterial community diversity in rhizosphere soil aggregates, significantly improving the bacterial community diversity in microaggregates in particular (Table S2). The results of the β NTI further indicated that the changes in bacterial communities in the microaggregates in the Z23 and Z39 treatment groups were homogeneously dispersed (Figure 7b), meaning that the influence of the exogenous inoculation of functional strains on the bacterial community structure was significant and this influence was a dominant random process. For all treatments, Actinobacteria, Proteobacteria, Chloroflexi, Acidobacteriota, Firmicutes, Myxococcota, Bacteroidota, Gemmatimonadota, and Planctomycetota were the dominant phyla, with a total relative abundance of more than 98% (Figure 7c). Compared with the CK treatment, inoculation with Z23 and Z39 increased the relative abundances of Proteobacteria, Firmicutes and Bacteroidota, and decreased those of Actinobacteria and Chloroflexi in the microaggregates (Figure 7c). Moreover, strains Z23 and Z39 also increased the relative abundances of *Rhizobiales*, *Gaiellales*, *Bacillales*, and *Burkholderiales* in the microaggregates (Figure 7d).

### 3.9. Functional Bacteria in Soil Aggregates

**Figure 8:**
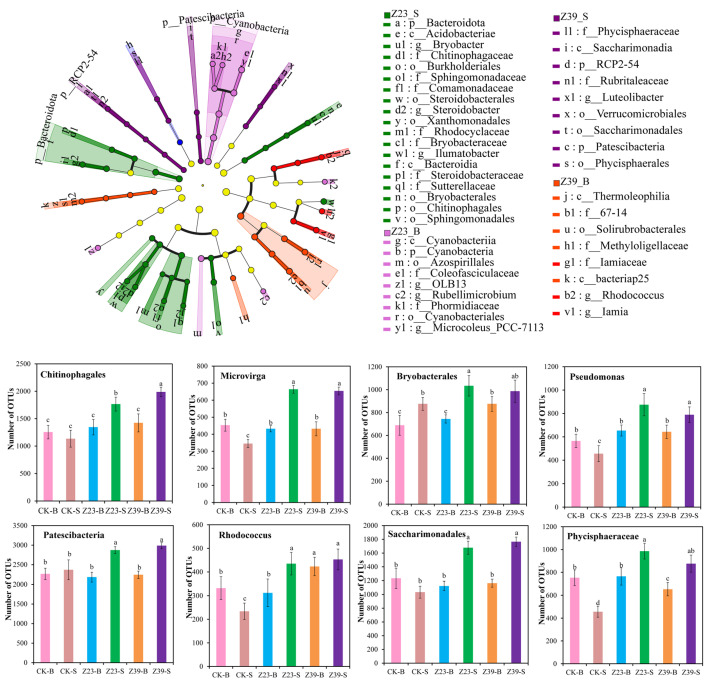
LEfSe analysis based on OTUs between the six treatments and the number of OTUs of typical strains. Bars indicated by the same letter were not significantly (*p* > 0.05) different according to Tukey’s test.

The exogenous inoculation of bacterial strains may have an impact on the indigenous functional bacterial community in the soil. The cladogram (Figure 8) showed that the key bacterial groups mainly included p__Bacteroidota, c__Acidobacteriae, g__Bryobacter, f__Sphingomonadaceae, o__Sphingomonadales, and o__Chitinophagales in the Z23-S treatment and c__Cyanobacteriia, o__Azospirillales, g__Rubellimicrobium, and g__Microcoleus__PCC-7113 in the Z23-B treatment. The cladogram also showed that the key bacterial groups mainly included f__Phycisphaeraceae, c__Saccharimonadia, g__Luteolibacter, o__Saccharimonadales, p__Patescibacteria, and o__Phycisphaerales in the Z39-S treatment and c__Thermoleophilia, o__Solirubrobacterales, f__Methyloligellaceae, and g__Rhodococcus in the Z39-B treatment (Figure 8). In addition, compared to the macroaggregates, the microaggregates contained a greater variety of key and functional bacteria, indicating that the exogenous inoculation of EPS-producing bacteria had a more significant impact on the bacterial community structure in the microaggregates and could mobilize more types of bacteria to participate in the detoxification of heavy metals. According to the literature, *Phycisphaeraceae* and *Patescibacteria* have the ability to immobilize heavy metals [41,42]. *Saccharimonadales* participates in glucose metabolism and N cycling [43]. *Chitinophagales* has the ability to dissolve P and promote plant growth [44]. *Bryobasterales* mediates iron (Fe) cycling and Cd fixation in rhizosphere soil [45,46]. These results indicated that inoculation with strains Z23 and Z39 altered the structure of the functional bacterial community and enriched strains with heavy metal-resistant, plant growth-promoting, and heavy metal-immobilizing properties in the rhizosphere soil, thereby inhibiting the absorption of Cd by lettuce.

### 3.10. Correlation Analysis

**Figure 9:**
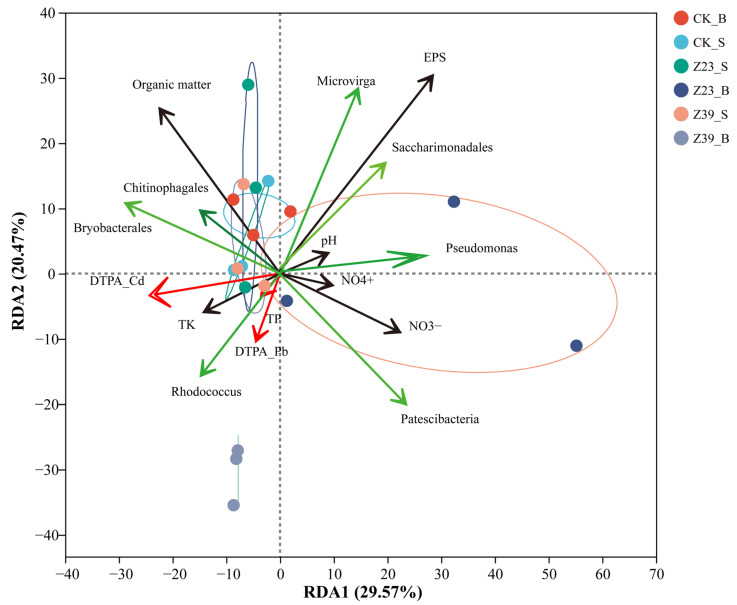
RDA of DTPA-extractable Cd and Pb contents and soil quality in the rhizosphere soils of lettuce.

Spearman correlation analysis and RDA revealed the key factors affecting the available heavy metal content in the rhizosphere soil (Figure 9). The effects of strains Z23 and Z39 on the total potassium (TK), total phosphorus (TP), ammoniacal nitrogen (NO_4_^+^), nitric nitrogen (NO_3_^−^), and EPS contents in the rhizosphere soils of the lettuce are detailed in Table S3. The results showed that the DTPA-Cd and Pb contents were strongly negatively correlated with the pH, NO_4_^+^, NO_3_^−^, and EPS levels (r = −0.534 to −0.905, *p* < 0.05) in the rhizosphere soil (Table S4). Furthermore, the DTPA-Cd and Pb contents presented a strong negative correlation with the abundance of *Patescibacteria*, *Saccharimonadales*, *Microvirga*, and *Pseudomonas* (r = −0.754 and −0.846, *p* < 0.05) (Table S4). The results indicated that the EPS content and the bacterial community associated with heavy metal resistance and N cycling (*Patescibacteria*, *Saccharimonadales*, *Microvirga*, and *Pseudomonas*) in microaggregates were key factors affecting the available heavy metal content in soil.

## 4. Discussion

Heavy metal-immobilizing bacteria are types of microorganisms that have been extensively studied for their ability to fix heavy metals and inhibit their absorption by crops [47,48]. The mechanisms of heavy metal immobilization used by these bacteria mainly include surface adsorption, intracellular enrichment, extracellular adsorption and chelation, biological precipitation, and oxidation–reduction [49,50]. There are multiple pathways for bacteria to repair heavy metals in the soil, among which the pathway of fixing and precipitating heavy metals through exopolysaccharides has been shown to have great potential [51]. Exopolysaccharide-producing bacteria immobilize heavy metals in solution and soil by secreting exopolysaccharides, thereby reducing the mobility of heavy metals [52]. In our study, two exopolysaccharide-producing bacteria, *Bacillus* sp. Z23 and Z39, were isolated from heavy metal-contaminated soil and exhibited high efficiency in adsorbing Cd and Pb and secreting exopolysaccharides. In solutions with a Cd concentration of 5 mg L^−1^ and a Pb concentration of 10 mg L^−1^, the adsorption and removal rates for strains Z23 and Z39 were above 90% for both, indicating that these two strains can efficiently immobilize Cd and Pb. Karthik et al. [53] isolated two exopolysaccharide-producing bacteria, AR6 and AR8, from the rhizosphere of kidney beans and demonstrated their good biosorption of chromium (Cr), reducing the accumulation of Cr in the roots and shoots. Cd, Cr, and Cu stimulated *Bacillus* sp. S3 to produce exopolysaccharides and enhanced its ability to adsorb and detoxify heavy metals [54]. Microbial adsorption occurs passively and in an independent metabolic manner, and both live and dead bacteria can absorb heavy metals through several physical and chemical mechanisms, such as ion exchange, complexation, precipitation, and chelation [55]. Even under low-metal-ion conditions, their ability to absorb ions is enhanced due to their excellent sensitivity, accumulation rate, and extent.

Negatively charged cell surfaces and exopolysaccharides bind to positively charged metal ions to fix heavy metals [56]. For example, the acetyl amino (-NH_2_) group of chitin; the structural polysaccharide of fungi; the amine (-NH_3_), sulfhydryl (-SH), and carboxylic (-COOH) groups in proteins; the phosphodiester (-COOP), phosphate (PO_4_^3−^), and hydroxyl (-OH) groups in polysaccharides; and other abundant active functional groups and non-carbohydrate substituents give the polymer an overall negative charge [57]. The XRD and XPS results indicated that strains Z23 and Z39 not only induced the mineralization and precipitation of Cd and Pb to form Fe_2_Pb(PO_4_)_2_, Cd_2_(PO_4_)_2_, and Pb_2_O_3_, but also played an important role in the adsorption and immobilization of Cd and Pb through the secretion of exopolysaccharides. Iyer et al. [58] studied the tolerance of the marine bacterium *Enterobacter cloaceae* (which produces extracellular polysaccharides) to Cr(VI), and found that the strain not only has tolerance to Cr(VI), but also strong Cr removal ability. Studies have shown that polysaccharide-producing bacteria reduce the bioavailability of heavy metals in soil, alleviate stress conditions, and promote plant growth and health [20]. *Agrobacterium tumefaciens* F2 was shown to have high adsorption ability for heavy metals such as Pb^2+^, Cd^2+^, and nickel (Ni^2+^), with the best adsorption effect for Pb^2+^. The adsorption mechanism was mainly due to the combination of heavy metals with functional groups such as C=O in carboxyl groups and C-O-C in sugar derivatives. Amino-acid-based proteins in EPS also adsorb heavy metals to some extent [59]. The protein components in extracellular polysaccharides have abundant -COOH, -OH, NH_3_^-^, and PO_4_^3-^ groups, with the highest adsorption capacity for Pb^2+^ [60]. Two polysaccharide-producing bacteria, *Bacillus gibsonii* PM11 and *Bacillus xiamenensis* PM14, were found to improve the nutrient utilization rate of flax and promoted its growth by alleviating metal stress [61]. In this study, strains Z23 and Z39 increased the dry weight of the lettuce roots and leaves, indicating that these strains have the ability to alleviate the toxicity of Cd and Pb in lettuce and promote the growth of various lettuce tissues. The polysaccharide-producing bacteria not only increased the content of extracellular polysaccharides and proteins in the soil, but also had the function of passivating plants from absorbing heavy metals [62]. Morillo Pérez et al. [63] showed that polysaccharides secreted by *Paenibaillus jamilae* could adsorb Cu(II), Ni(II), and Pb(II), and found a maximum adsorption capacity of 303 mg g^−1^ for Pb(II). The polysaccharide-producing microorganisms produced extracellular polysaccharides, provided the necessary C and N sources for plants, and also showed good application prospects in the remediation of heavy metal pollution in soil. The exopolysaccharides secreted by exopolysaccharide-producing bacteria released phosphate ions and induced heavy metals to form precipitates, thus reducing the availability of heavy metals [64]. In addition, these bacteria also secreted some plant growth hormones, promoted plant growth, and prevented plants from absorbing heavy metals [14]. In this study, the polysaccharide-producing bacteria Z23 and Z39 reduced the content of Cd and Pb in wheat roots and leaves, indicating that these strains have the ability to inhibit the absorption of heavy metals by lettuce and can be used as heavy metal passivators in further research.

Soil aggregates are the basic components of the soil structure. Due to the different composition and properties of aggregates with different particle sizes, their characteristics in terms of the adsorption of environmental pollutants are also different [65]. The behavior of heavy metals in soil is largely restricted by the distribution of aggregates, which affect their bioavailability and migration ability. Research has shown that there is a significant relationship between the enrichment of heavy metals and the size of aggregates, with smaller aggregates having a larger specific surface area and higher organic matter content, which is beneficial for the enrichment of heavy metals [66]. Regulating the size of the soil aggregates is important for studying the distribution and bioavailability of heavy metals. By adding exopolysaccharide-producing bacteria to the soil, the size of the soil aggregates can be changed, thus affecting the absorption of heavy metals by crops [67]. Wu et al. [68] found that *Bacillus amyloliquefaciens* HYD-B17, *Bacillus licheniformis* HYTAPB18, and *Bacillus subtilis* RMPB44 with high exopolysaccharide production improved the stability of soil aggregates. Our study showed that the polysaccharide-producing bacteria Z23 and Z39 increased the proportion of microaggregates in the rhizosphere soil of lettuce, thereby enhancing the ability of the soil to adsorb and fix Cd and Pb. In addition, exopolysaccharide-producing bacteria were introduced into the soil to bind the soil particles together and form stable aggregates. The stability of soil aggregates is a symbolic factor for excellent soil structure. Therefore, improving the stability of aggregates improves the retention of soil water and fertilizer [67]. Exopolysaccharide-secreting bacteria are of great significance for the formation and stability of soil aggregates and the improvement of the soil structure [69]. In recent decades, scholars have conducted a significant amount of in-depth research on the mechanism of soil microorganisms in the formation and stability of soil aggregates [70]. Current studies have mainly focused on the influence of fungi on the formation of soil aggregates, because fungi can physically intertwine soil particles in aggregates through mycelium and connect them through the organic cementing of metabolites [71]. However, because the mycelium is easily decomposed by other microbial activities and forms an unstable aggregate structure, it does not last long in the soil. Therefore, more scholars began to study the effects of bacteria and their exopolysaccharides on the formation of soil aggregates [72]. The results showed that extracellular polysaccharides produced by bacteria are more beneficial to the formation and stability of soil aggregates than bacteria. In our study, inoculating Z23 and Z39 significantly increased the content of polysaccharides in the soil aggregate. The content of polysaccharides in microaggregates was significantly higher than that in large aggregates, indicating that microaggregates play an important role in immobilizing heavy metals.

Soil aggregates are the main environment in which soil microorganisms are active. Soil aggregates provide ecological niches for different microbial communities with specific classifications, functions, and ecological adaptability to environmental disturbances [73]. Therefore, soil aggregates can determine the resources available to soil microorganisms through oxygen diffusion, water flow, the accessibility of organic matter, and nutrient availability [74]. The chemical composition, organic matter decomposition ability, cation exchange capacity, surface reactivity, and adsorption characteristics of different particle size aggregates also vary; therefore, there are differences in the soil microbial communities living in them. Soil microorganisms can form and stabilize soil aggregates by binding clay particles together through their own activities and extracellular polysaccharides produced by certain fungal hyphae. In our study, inoculation with polysaccharide-producing bacteria increased the proportion of microaggregates in the rhizosphere soil of lettuce. More importantly, compared to macroaggregates, microaggregates had a more significant impact on the bacterial community structure. The vast majority (90%) of soil microorganisms are related to microaggregates, with the vast majority (70%) living within microaggregates and very few microorganisms in direct contact with each other. Only a small portion (<10%) of microorganisms are located on the surface of large aggregates exposed to large pores. Some scholars have found that fungi in soil, especially arbuscular mycorrhizal fungi, produce hyphae that are mainly related to the formation and stability of large aggregates [75]. Some sand particles in aggregates are only connected by fungal hyphae, while soil bacteria are mainly involved in the formation and stability of microaggregates [76]. In our study, inoculation with strains Z23 and Z39 altered the structure of the functional bacterial community and enriched strains with heavy metal-resistant, plant growth-promoting, and heavy metal-immobilizing properties in the rhizosphere soil, thereby inhibiting the absorption of Cd and Pb by lettuce. Thus, strains Z23 and Z39 have shown great potential for the remediation of excess Cd and Pb in farmland, collaborating with indigenous microbial communities in the soil to regulate the structure of rhizosphere soil aggregates, allowing heavy metals to precipitate into soil microaggregates, and reducing the mobility and available content of Cd and Pb, thereby avoiding the crop root absorption of heavy metals and ensuring food safety. However, functional strains are susceptible to external environmental influences and their functions may be unstable. Further research on the stability of strain functions and the validation of field effects is needed.

## 5. Conclusions

Two bacterial strains, Z23 and Z39, with high exopolysaccharide production were screened from heavy metal-contaminated soil. Both strains belong to the genus *Bacillus* sp. Strain Z23 had a removal rate of 88.6% for Cd and 93.2% for Pb. Strain Z39 had a removal rate of 92.3% for Cd and 94.4% for Pb. Strains Z23 and Z39 reduced the content of Cd and Pb in solution, inducing the formation of precipitates such as Fe_2_Pb(PO_4_)_2_, Cd_2_(PO_4_)_2_, and Pb_2_O_3_. Moreover, the polysaccharides secreted by the strains also participate in the adsorption of heavy metals. Pot experiments showed that strains Z23 and Z39 increased the dry weight and antioxidant enzyme activity of lettuce roots and leaves, while reducing the content of Cd and Pb by 40.1–61.7%. The strains increased the proportion of microaggregates (<0.25 mm) and exopolysaccharide content in the rhizosphere soil, reduced the content of available Cd and Pb, and prevented the absorption of heavy metals by lettuce. Moreover, the inoculation with strains Z23 and Z39 altered the structure of the functional bacterial community in the microaggregates, thereby inhibiting the absorption of Cd and Pb by lettuce. Our results provide strain resources and a theoretical basis for microbial remediation in heavy metal-contaminated fields.

## Data Availability

All data are included in the article and Supplementary Materials.

## Supplementary Materials

The following supporting information can be downloaded at https://www.mdpi.com/article/10.3390/microorganisms12112112/s1: Figure S1: Phylogenetic tree constructed using the Neighbor Joining method on the basis of 16S rDNA sequences of strains. Table S1: Promoting characteristics and heavy metal resistance of functional strains. Table S2: Estimation of bacterial community diversity. Table S3. Determination of physical and chemical indicators of lettuce rhizosphere soil. Table S4: Spearman correlation analysis of physical and chemical properties of lettuce rhizosphere soil.
