# Supplementary material for: Exopolysaccharide-Producing Bacteria Regulate Soil Aggregates and Bacterial Communities to Inhibit the Uptake of Cadmium and Lead by Lettuce
Source: Microorganisms. 2024 Oct 22;12(11):2112. doi: 10.3390/microorganisms12112112 (PMC11596253; doi:10.3390/microorganisms12112112)
Supplement: Supplementary file 1 [file microorganisms-12-02112-s001.zip › microorganisms-3228655-supplementary.pdf]

Supplementary Materials for

Exopolysaccharide-Producing Bacteria Regulate Soil Aggregates and Bacterial Communities to Inhibit the Uptake of Cadmium and Lead by Lettuce

Heyun Zhang [1], Ke Wang [1], Xinru Liu [1], Lunguang Yao [2], Zhaojin Chen [1,*] and Hui Han [1,2,*]

[1] Collaborative Innovation Center of Water Security for Water Source Region of Mid-route Project of South-North Water Diversion of Henan Province, Nanyang Normal University, Nanyang 473061, China; zhangheyun66@163.com (H.Z.); m13080182307@163.com (K.W.); 17651962022@163.com (X.L.)

[2] Henan Field Observation and Research Station of Headwork Wetland Ecosystem of the Central Route of South-to-North Water Diversion Project, Nanyang Normal University, Nanyang 473061, China; lunguangyao@nynu.edu.cn

* Correspondence: zhaojin_chen@163.com (Z.C.); hanhui2018@nuynu.edu.cn (H.H.)

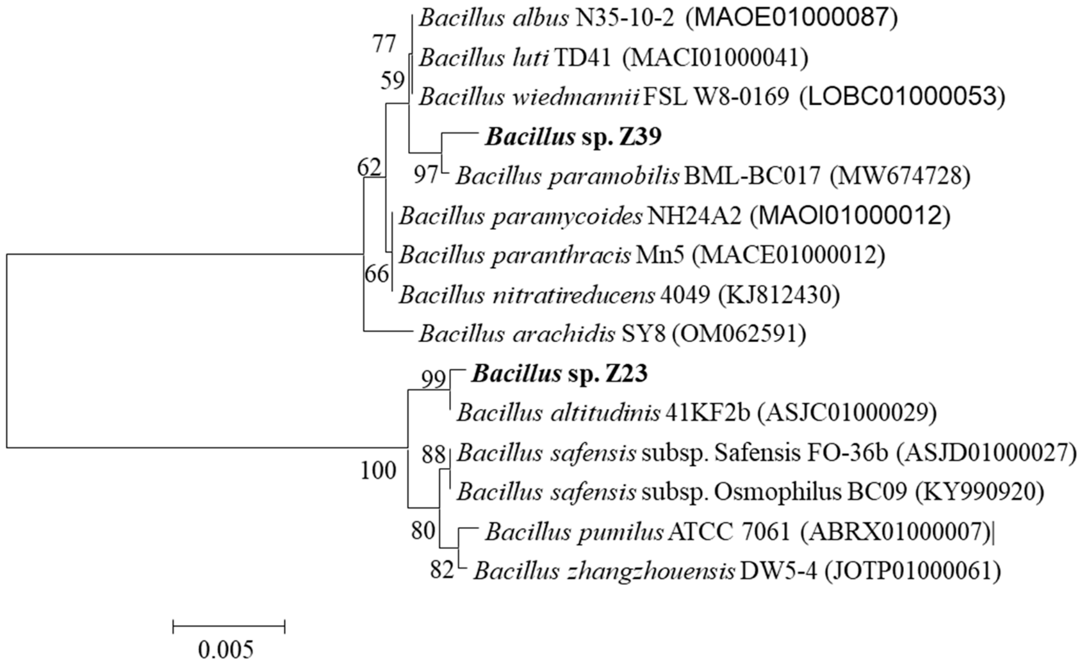

Figure S1. Phylogenetic tree constructed by the Neighbor Joining method on the basis of 16S rDNA sequences of strains

Table S1. Promoting characteristics and heavy metal resistance of functional strains

	Z23	Z39
IAA (mg L^{-1})	57.5	69.2
Siderophore	+++	+++++
ACC deaminase	+	+
Lethal concentration of Pb (mg L^{-1})	400	500
Lethal concentration of Pb (mg L^{-1})	1800	2000
Gram stain	+	+

Table S2 Estimation of bacterial community diversity

Sample	Sobs	Chao I	Shannon	Simpson	Ace	Coverage
CK-B	3511±131a	4834±121a	6.812±0.018a	0.0033±0.0002b	4823±153a	0.9705±0.0029a
CK-S	3514±98a	4765±154a	6.815±0.027a	0.0034±0.0002b	4787±106a	0.9706±0.0024a
Z23-B	3443±154a	4654±187a	6.761±0.033a	0.0034±0.0002b	4687±1326a	0.9717±0.0039a
Z23-S	3387±129a	4687±176a	6.622±0.043b	0.0048±0.0005a	4674±129a	0.9678±0.0021a
Z39-B	3423±174a	4665±245a	6.781±0.033a	0.0035±0.0002b	4697±326a	0.9719±0.0039a
Z39-S	3409±164a	4678±187a	6.618±0.043b	0.0049±0.0005a	4665±129a	0.9674±0.0021a

Large aggregate soil (CK-B) and microaggregates soil (CK-S) in the CK treatment group, large aggregate soil (Z23-B) and microaggregates soil (Z23-S) in the Z23 treatment group, and large aggregate soil (Z39-B) and microaggregates soil (Z39-S) in the Z39 treatment group. Means within the same column that are followed by the same letter are not significantly different at $P < 0.05$ based on one-way ANOVA

Table S3. Determination of physical and chemical indicators of lettuce rhizosphere soil

	pH	organic matter (mg kg^{-1})	Total K (g kg^{-1})	Total P (g kg^{-1})	NO_4^- content (mg kg^{-1})	NO_3^+ content (mg kg^{-1})
CK-B	6.93±0.0d	27.4±0.13a	1.34±0.09a	1.45±0.13a	423±8e	231±11c
CK-S	6.91±0.0d	26.2±0.12a	1.27±0.11a	1.34±0.11a	421±11e	222±8c
Z23-B	6.98±0.0c	27.5±0.09a	1.26±0.07a	1.41±0.16a	657±13c	324±12a
Z23-S	7.09±0.0a	26.8±0.11a	1.27±0.08a	1.38±0.06a	798±22a	335±11a
Z39-B	6.98±0.0c	27.4±0.16a	1.26±0.11a	1.42±0.09a	617±9d	287±6b
Z39-S	7.05±0.0b	27.1±0.18a	1.28±0.16a	1.43±0.17a	715±11b	321±9a

Large aggregate soil (CK-B) and microaggregates soil (CK-S) in the CK treatment group, large aggregate soil (Z23-B) and microaggregates soil (Z23-S) in the Z23 treatment group, and large aggregate soil (Z39-B) and microaggregates soil (Z39-S) in the Z39 treatment group. Means within the same column that are followed by the same letter are not significantly different at $P < 0.05$ based on one-way ANOVA.

Table S4. Spearman correlation analysis of physical and chemical properties of lettuce rhizosphere soil.

	DTPA-Pb	EPS	NO_4^+	NO_3^-	pH	TK	TP	Organic matter	Bryobacterales	Chitinophagale	Rhodococcus	Patescibacteria	Pseudomonas	Saccharimonadales	Microvirga
DTPA-Cd	0.354	-0.905	-0.822	-0.654	-0.875	0.187	0.257	0.367	0.347	0.214	0.421	-0.864	-0.785	-0.812	-0.831
DTPA-Pb		-0.814	-0.823	-0.534	-0.843	0.278	0.374	0.425	0.289	0.247	0.357	-0.788	-0.754	-0.774	-0.792
EPS			0.456	0.287	0.397	-0.354	-0.284	-0.436	-0.147	-0.234	-0.351	0.321	0.216	0.364	0.261
NO_4^+				0.416	0.317	-0.367	-0.187	-0.258	-0.361	-0.164	-0.367	0.245	0.194	0.253	0.421
NO_3^-					0.287	-0.487	-0.184	-0.325	-0.174	-0.421	-0.254	0.268	0.363	0.285	0.365
pH						-0.265	-0.147	-0.214	-0.258	-0.364	-0.267	0.421	0.164	0.3644	0.254
TK							0.234	0.352	0.153	0.258	0.234	-0.365	-0.241	-0.342	-0.265
TP								0.321	0.356	0.294	0.164	-0.362	-0.195	-0.361	-0.275
Organic matter									0.253	0.365	0.326	-0.362	-0.174	-0.269	-0.362
Bryobacterales										0.352	0.212	-0.132	-0.324	-0.193	-0.342
Chitinophagale											0.194	-0.234	-0.353	-0.298	-0.267
Rhodococcus												-0.384	-0.538	-0.429	-0.298
Patescibacteria													-0.328	-0.428	-0.324
Pseudomonas														-0.359	-0.427
Saccharimonadales															-0.325
